# Assessment of macular structures and vascular characteristics in highly myopic anisometropia using swept-source optical coherence tomography angiography

**DOI:** 10.3389/fphys.2022.918393

**Published:** 2022-08-15

**Authors:** Xin Wang, Yanhui Chen, Zhiyang Wang, Haoru Li, Qing He, Hua Rong, Ruihua Wei

**Affiliations:** ^1^ Tianjin Key Laboratory of Retinal Functions and Diseases, Tianjin Branch of National Clinical Research Center for Ocular Disease, Eye Institute and School of Optometry, Tianjin Medical University Eye Hospital, Tianjin, China; ^2^ Tangshan Eye Hospital, Tangshan, China

**Keywords:** anisometropia, high myopia, optical coherence tomography angiography, macular structures, vascular characteristics

## Abstract

**Purpose:** This study aimed to explore the macular structures and vascular characteristics of more myopic (MM) and contralateral eyes with highly myopic anisometropia.

**Methods:** Comprehensive ophthalmic examinations were performed for 33 patients with highly myopic anisometropia. Macular structures (total retinal layer [TRL], ganglion cell and inner plexiform layer [GCIPL], inner nuclear layer [INL], outer retinal layer [ORL], nerve fiber layer [NFL], choroidal layer [CHL]) and vascular characteristics (superficial vascular complex density [SVD], deep vascular complex density [DVD], choriocapillaris perfusion area [CCPA]) were assessed using swept-source optical coherence tomography (SS-OCT) and OCT angiography (OCTA). Macular structures and vascular characteristics of each subregion were compared to those of the Early Treatment of Diabetic Retinopathy Study (ETDRS).

**Results:** With highly myopic anisometropia, the thicknesses of the TRL, GCIPL, INL, and ORL in MM eyes were smaller than those in contralateral eyes in at least one quadrant of the perifoveal and parafoveal circles (all *p* < 0.05), with no changes in the foveal and temporal quadrants of perifoveal regions (all *p* > 0.05). A thicker NFL (*p* = 0.018) was found in MM eyes than in contralateral eyes in the superior perifoveal quadrant. The CHL (all *p* < 0.05) in MM eyes was thinner in all regions than in the contralateral eyes according to the ETDRS. There were no statistical differences in the SVD, DVD, and CCPA of MM and contralateral eyes (all *p* > 0.05).

**Conclusion:** All retinal layers, except the NFL, tended to be thinner in all subregions, except the temporal perifoveal and foveal quadrants in MM eyes, and choroidal thickness was thinned in all areas.

## Introduction

Myopia has become a global public health concern and will affect approximately 50% of the global population by 2050, and high myopia will affect approximately 10% of the global population (Holden et al., 2016). High myopia is associated with a lengthening of the axis length (AL), changes in the retinal and choroidal structures, and microcirculation. Macular degeneration with high myopia is a major risk of vision loss and leads to irreversible vision damage and significant economic and social consequences (Naidoo et al., 2019; Ohno-Matsui and Jonas, 2019). Therefore, knowledge of the structures and vascular characteristics of the macula can assist with the analysis of the changes in the macular region occurring with high myopia and allow for the exploration of the pathogenesis of macular degeneration.

Optical coherence tomography (OCT) is widely used for clinical examination of macular retinal morphology (Breher et al., 2020). Several studies have compared the changes in retinal thickness with myopic severity; however, those studies evaluated few layers of the retinal structure or reported inconsistent conclusions. Several studies have found that as the AL increases with high myopia, the peripheral area becomes thinner and the fovea remains the same (Jonas et al., 2016; Del-Prado-Sanchez et al., 2021). Lim et al. (Lim et al., 2005) found that the mean thickness of the macula does not change with myopic severity, but that the parafovea retina becomes thinner and the fovea becomes thicker. Liu et al. (Liu et al., 2015) reported that some layers of the retinal thickness were thicker in the high myopia group than in the control group with normal eyes. Choroidal thinning can predict myopia progression (Fontaine et al., 2017; Jin et al., 2019). Because of its greater penetration, swept-source OCT (SS-OCT) has been widely used during retinal and choroidal thickness studies (Dastiridou et al., 2017). Using SS-OCT and OCT angiography (OCTA), several researchers have conducted qualitative and quantitative studies of the retinal and choroidal vascular characteristics of patients with myopia (Fan et al., 2017; Mo et al., 2017); however, the results have been controversial. Yang et al. (Yang et al., 2020) concluded that macular blood density tends to decrease in patients with high myopia without significant degenerative lesions. However, Mo et al. (Mo et al., 2017) showed that the retinal blood flow density was not altered in patients with high myopia.

The varying results of previous studies may have occurred because of the different study populations or magnifications caused by the AL, which are not routinely corrected using commercially available instruments. Therefore, we corrected for ocular magnification using the AL in our study. Moreover, the variability in the results of these studies may be related to factors such as AL, age, sex, and segmentation method (Wang et al., 2021). To more accurately evaluate the changes in macular structures and vascular characteristics caused by the AL with high myopia, patients with highly myopic anisometropia were recruited for our study to minimize some variables, such as environment, heredity, age, sex, and other factors, that may affect the results.

Relatively few studies have evaluated the macular structures and vascular characteristics associated with highly myopic anisometropia. This study aimed to investigate the differences in macular structures and vascular characteristics of more myopic (MM) and contralateral eyes with highly myopic anisometropia.

## Methods

### Subjects

This observational, cross-sectional study included 66 eyes of 33 patients with highly myopic anisometropia from May 2021 to February 2022 at the Eye Hospital of Tianjin Medical University. All examinations were conducted according to the tenets of the Declaration of Helsinki and were approved by the Ethics Committee of the Eye Hospital of Tianjin Medical University. Written informed consent was obtained from all subjects.

### Biomechanical parameters

We recruited patients with highly myopic anisometropia for our study. All patients underwent a comprehensive eye examination. Complete anterior and posterior examinations were performed using a slit lamp biomicroscope (TSL-5; Gaush, Wenzhou, China). Best-corrected visual acuity (BCVA) and refractive status were examined by an experienced optometrist using a phoropter (VT-10; Topcon, Japan). A non-contact tonometer (CT-1; Topcon, Japan) was used to measure intraocular pressure. Fundus photographs were obtained using mydriatic fundus photography (CR-2, Canon, Japan). A non-contact biometer (Lenstar LS-900; Haag-Streit AG, Berne, Switzerland) was used to measure the AL. Refractive errors were converted into standard errors (SEs). The SE algorithm included the spherical diopter plus half of the cylindrical diopter.

Myopic anisometropia is a condition in which the refractive state of both eyes is asymmetrical, usually because of differences in the AL of the two eyes. The eyes develop two different states at a spherical equivalent refraction (SER; sphere +1/2 cylinder) of ≥1.00 D between the two eyes (O'Donoghue et al., 2013). Therefore, our inclusion criterion was a diagnosis of highly myopic anisometropia. The SER of both eyes was ≤–6.00. The SER difference between the MM and contralateral eyes was ≥1 D. The difference in the eye AL was ≥0.3 mm. The exclusion criteria were as follows: visual acuity examination using the Snellen eye chart, BCVA <20/25; highly myopic fundus changes other than tessellated fundus appearance or diffuse chorioretinal atrophy changes, such as patchy atrophy, neovascularization, or traction retinopathy; eye diseases other than high myopia, such as glaucoma or other vitreoretinal diseases; systemic disease causing retinal and choroidal changes, such as diabetes; and history of ophthalmic surgery or trauma. The signal strength of the OCT/OCTA image was <6. The images were not centered on the central fovea.

### Swept-source optical coherence tomography (SS-OCT)/OCT angiography image acquisition and analysis

The SS-OCT/OCTA system (VG200S; SVision Imaging, Henan, China) was used during this study to quantify the macular structures and vascular characteristics of all enrolled eyes. This device was equipped with an SS laser and an eye tracking device that eliminated eye movement artifacts. The central wavelength was 1,050 nm (full width range, 990–1,100 nm), and the scan rate was 200,000 A-scans per second. The axial and lateral resolutions of the OCT tool were 5 and 13 μm, respectively.

**FIGURE 1:**
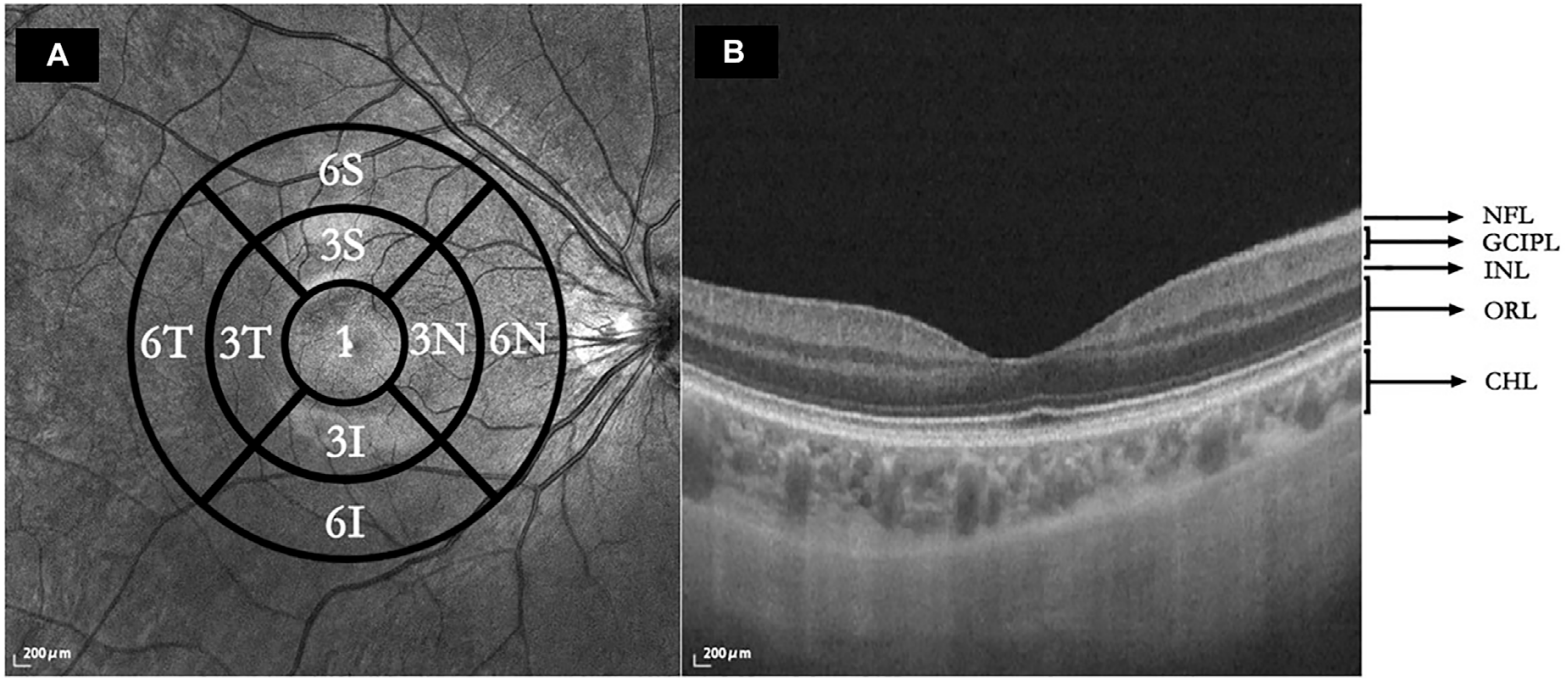
**(A)** Macular regions according to the Early Treatment of Diabetic Retinopathy Study. 1: central foveal circle; 3: parafoveal circle; 6: perifoveal circle; T: temporal; N: nasal; S: superior; I: inferior. **(B)** Segmentation of five intraretinal layers and choroidal layer. Using SS-OCT/OCTA, the cross-sectional image was centered on the fovea retina during the horizontal scan and segmentation of the intraretinal and choroidal layers was performed. NFL: nerve fiber layer; GCIPL: ganglion cell layer and inner plexiform layer; INL: inner nuclear layer; ORL: outer plexiform layer and outer nuclear layer and photoreceptor layer and retinal pigment epithelium (RPE)/Bruch’s membrane complex layer; CHL: choroid layer.

Macular intraretinal thickness, choroidal thickness, retinal vascular density, and choriocapillaris perfusion area (CCPA) were obtained using a raster scan protocol and 512 A-scans × 512 B-scans. The scan cube was 6 mm × 6 mm and was centered on the fovea. The scan depth was 6 mm. Bennett’s formula was used to adjust the image magnification caused by AL. According to the grid of the Early Treatment of Diabetic Retinopathy Study (ETDRS), macular images are classified into three concentric circles centered on the fovea. The 1-, 3-, and 6-mm diameters comprised the foveal, parafoveal, and perifoveal circles, respectively. The parafoveal and perifoveal circles are further subdivided into four subfields: superior, temporal, inferior, and nasal subfields (Figure 1A).

**FIGURE 2:**
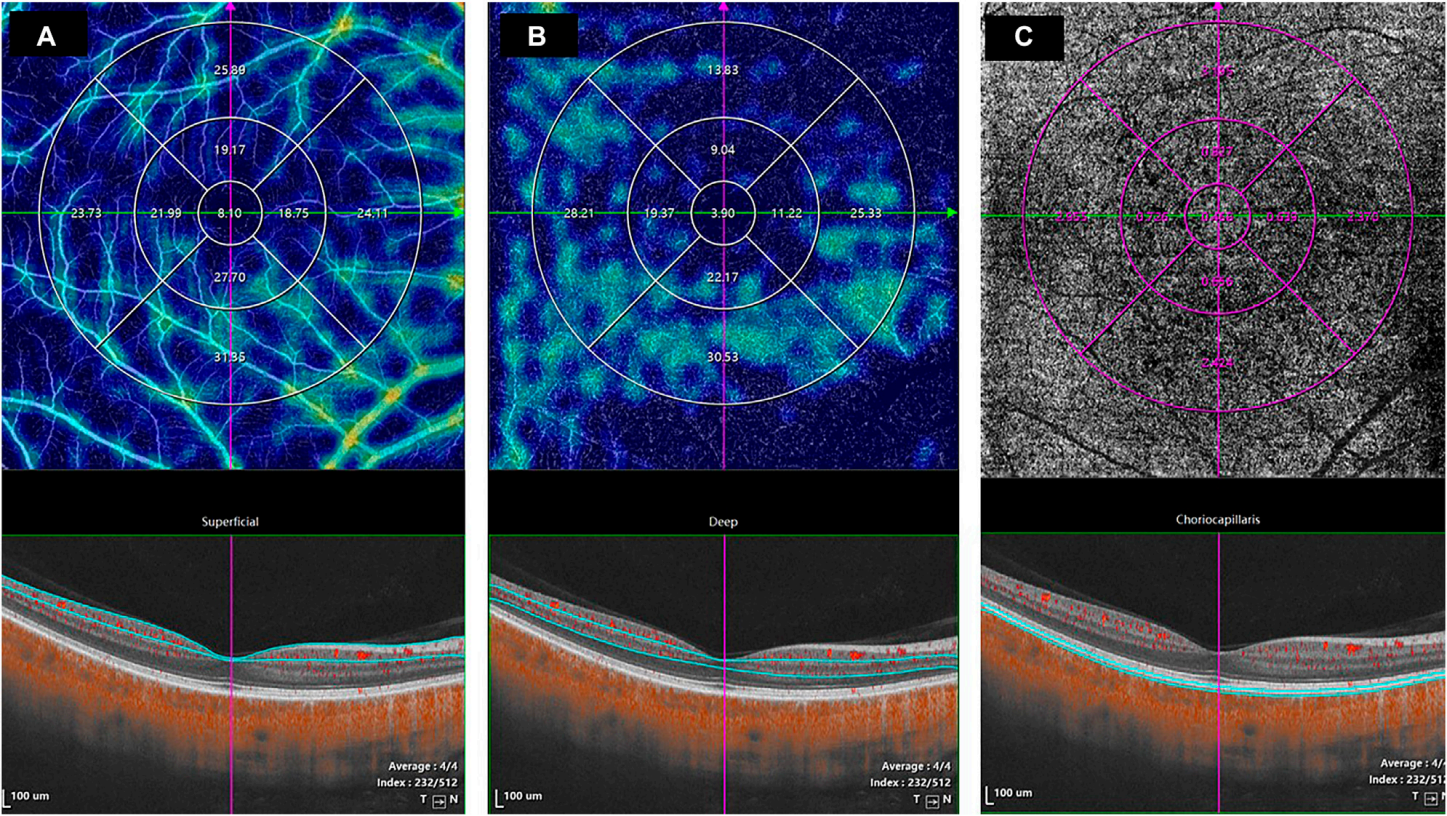
The values of the microcirculation parameters were calculated automatically by the system. The top *en face* images are intensity projections of the regions selected by the blue lines in the bottom images. **(A)** The retinal superficial vascular complex was automatically defined by the system as microvessels found 5 μm above the inner limiting membrane to one-third of the interface of the ganglion cell layer and inner plexiform layer (GCIPL). **(B)** The retinal deep vascular complex was automatically defined by the system as one-third of the interface of the GCIPL to 25 μm below the lower border of the outer plexiform layer. **(C)** The choriocapillaris was automatically defined by the system as the microvasculature from 10 μm above the RPE-Bruch’s membrane complex to 25 μm below it.

The horizontal scans of the macular tomography map for each subject were selected to segment the intraretinal and choroidal layers (CHLs) into five subfields, and the thickness of each subfield was calculated using built-in software. The total retinal layer (TRL) thickness was moved perpendicularly to the inner edge of the internal limiting membrane (ILM) and the outer edge of the retinal pigment epithelium (RPE)/Bruch’s membrane complex. The nerve fiber layer (NFL) thickness was moved perpendicularly from the inner to the outer edge of the nerve fiber layer. The ganglion cell-inner plexiform layer (GCIPL) thickness was moved perpendicularly from the inner edge of the ganglion cell layer to the outer edge of the inner plexiform layer (IPL). The inner nuclear layer (INL) thickness was moved perpendicularly from the inner to the outer edge of the INL. The thickness of the outer retinal layer (ORL) was measured from the inner edge of the outer plexiform layer (OPL) to the outer edge of the RPE/Bruch’s membrane complex. The CHL thickness was distanced perpendicularly from the outer edge of the RPE/Bruch’s membrane complex to the inner surface of the choroidoscleral border (Figure 1B). The retinal superficial vascular complex was automatically defined by the system as vessels observed 5 μm above the ILM to one-third of the interface of the GCIPL. Furthermore, the retinal deep vascular complex was automatically defined by the system as one-third of the interface of the GCIPL to 25 μm below the inner edge of the OPL. Additionally, the choriocapillaris was automatically defined by the system as the vasculature from 10 μm above to 25 μm below the RPE/Bruch’s membrane complex (Figure 2).

### Statistical analyses

Statistical analyses were performed using SPSS software (version 26.0, SPSS, IBM, Chicago). The normality of the data was tested using the Kolmogorov–Smirnov normality test. All continuous variables are expressed as mean ± standard deviation (SD). Paired t-tests were used to compare continuous variables of normal distributions between MM and contralateral eyes for ocular biometrics. Continuous variables non-normally distributed between the two groups were compared by Wilcoxon’s rank-sum tests. Statistical significance was set at *p* < 0.05.

## Results

### Demographics

A total of 33 (16 men and 17 women) subjects with highly myopic anisometropia were included in this study. The subjects’ average age was 30.15 (SD, ± 2.16) years. The SER of the MM eyes was –11.80 ± 3.39 D, which was lower than that of the contralateral eyes (–9.88 ± 3.22 D, *p* < 0.001). The AL of the MM eyes (28.23 ± 1.51 mm) was significantly longer than that of the contralateral eyes (27.45 ± 1.44 mm) (*p* < 0.001) (Table 1).

**TABLE 1:** Demographic and clinical characteristics of participants with highly myopic anisometropia

Parameter	More myopic eyes	Contralateral eyes	*P*
Age (years) ± SD	30.15 ± 2.16		
Sex, female/male (%)	17 (51.50)/16 (48.50)		
SE (D) ± SD	−11.80 ± 3.39	−9.88 ± 3.22	**<0.001** ^a^
AL (mm) ± SD	28.23 ± 1.51	27.45 ± 1.44	**<0.001** ^b^

a *p* value determined by the Wilcoxon signed-rank test.

b *p* value determined by the paired *t*-test.

Significant *p* values (*p* < 0.05) are in bold. D: diopter; SE: spherical equivalent; AL: axial length.

### Comparison of the intraretinal and choroidal layer thicknesses

The thicknesses of the nine different regions in each intraretinal and CHL are summarized in Table 2. The MM and contralateral eyes were also compared. No significant difference in the central foveal circle parameters of the GCIPL, INL, ORL, and NFL were observed (all *p >* 0.05). The TRL, INL, and ORL became thinner in the parafoveal circles in the MM eyes than in the contralateral eyes (all *p* < 0.05). The TRL, GCIPL, INL, and ORL became thinner in the perifoveal circles in the MM eyes than in the contralateral eyes (all *p* < 0.05), with no significant changes in the temporal quadrants of the perifoveal circles (all *p > 0.05*). The NFL was thicker in the MM eyes than in the contralateral eyes in the superior perifoveal quadrant (*p* = 0.018). The choroidal thickness in the MM eyes was thinner than that in the contralateral eyes in all areas according to the ETDRS (all *p* < 0.05) (Table 2).

**TABLE 2:** Comparison of the regional thickness of each retinal layer and choroidal layer of more myopic and contralateral eyes.

		MM	Contralateral	*P*
Total retinal layer (μm)	1	245.60 ± 23.86	248.81 ± 23.65	**0.010** ^b^
	3S	323.48 ± 19.54	328.44 ± 18.00	**<0.001** ^b^
	3T	309.00 ± 17.46	313.36 ± 18.64	**<0.001** ^b^
	3I	317.41 ± 18.88	320.96 ± 19.65	**0.016** ^b^
	3N	322.21 ± 20.65	324.68 ± 20.09	0.074**^b^**
	6S	286.73 ± 15.74	290.58 ± 15.62	**0.004** **^b^**
	6T	269.71 ± 14.15	270.75 ± 14.57	0.306**^b^**
	6I	267.81 ± 16.04	271.39 ± 17.88	**0.017** **^a^**
	6N	298.74 ± 18.32	304.46 ± 19.82	**<0.001** **^b^**
Ganglion cell-inner plexiform layer (μm)	1	25.81 ± 7.84	26.44 ± 8.05	0.3264**^b^**
	3S	88.39 ± 7.49	89.25 ± 7.36	0.062**^a^**
	3T	83.20 ± 9.54	84.90 ± 9.92	0.055**^a^**
	3I	86.25 ± 9.83	87.49 ± 8.10	0.249**^a^**
	3N	87.35 ± 8.73	85.60 ± 9.51	**0.046** **^a^**
	6S	59.19 ± 6.85	60.50 ± 5.69	**0.046** **^a^**
	6T	63.17 ± 9.50	62.77 ± 8.46	0.612**^b^**
	6I	53.54 ± 6.05	54.62 ± 6.58	0.141**^b^**
	6N	63.86 ± 8.11	65.83 ± 7.13	**0.022** **^a^**
Inner nuclear layer (μm)	1	26.21 ± 3.12	26.84 ± 3.33	0.110**^b^**
	3S	42.40 ± 3.28	43.78 ± 3.68	**<0.001** **^b^**
	3T	40.46 ± 3.34	41.31 ± 2.92	**0.015** **^b^**
	3I	42.38 ± 2.96	44.12 ± 3.65	**<0.001** **^b^**
	3N	42.49 ± 3.61	42.58 ± 3.75	0.805**^b^**
	6S	35.72 ± 1.87	36.79 ± 2.62	**0.004** **^b^**
	6T	38.26 ± 2.67	38.43 ± 2.22	0.778**^b^**
	6I	34.62 ± 1.99	35.41 ± 2.18	**0.019** **^b^**
	6N	38.82 ± 2.28	39.50 ± 2.55	0.163**^b^**
Outer retina layer (μm)	1	177.88 ± 18.46	179.81 ± 17.22	0.083**^b^**
	3S	162.89 ± 10.73	165.69 ± 11.14	**0.002** **^b^**
	3T	164.57 ± 10.70	166.22 ± 10.88	**0.040** **^b^**
	3I	158.72 ± 12.40	159.10 ± 11.77	0.607**^b^**
	3N	165.78 ± 11.19	169.27 ± 11.23	**<0.001** **^b^**
	6S	144.82 ± 10.29	147.77 ± 10.56	**<0.001** **^b^**
	6T	142.80 ± 9.50	143.72 ± 10.07	0.278**^b^**
	6I	133.45 ± 10.22	134.58 ± 11.27	0.165**^b^**
	6N	140.15 ± 10.71	143.64 ± 10.49	**<0.001** **^b^**
Nerve fiber layer (μm)	1	15.88 ± 2.92	15.83 ± 2.67	0.911**^b^**
	3S	29.87 ± 2.87	29.68 ± 2.64	0.729**^b^**
	3T	20.83 ± 3.89	21.10 ± 3.54	0.321**^a^**
	3I	30.12 ± 2.61	30.26 ± 3.42	0.964**^a^**
	3N	26.69 ± 4.19	27.05 ± 7.17	0.458**^a^**
	6S	47.29 ± 5.47	45.78 ± 4.86	**0.018** **^a^**
	6T	25.82 ± 5.50	25.99 ± 5.71	0.662**^a^**
	6I	46.50 ± 5.46	46.95 ± 5.84	0.448**^a^**
	6N	56.10 ± 7.31	55.76 ± 8.50	0.221**^a^**
Choroid layer (μm)	1	185.13 ± 76.62	221.54 ± 87.93	**<0.001** **^b^**
	3S	196.46 ± 78.57	234.81 ± 91.99	**<0.001** **^a^**
	3T	199.07 ± 80.93	233.53 ± 91.21	**<0.001** **^a^**
	3I	186.80 ± 75.86	218.45 ± 90.25	**<0.001** **^a^**
	3N	165.71 ± 66.59	199.05 ± 80.76	**<0.001** **^b^**
	6S	220.51 ± 89.80	253.89 ± 93.99	**<0.001** **^b^**
	6T	215.17 ± 77.26	246.13 ± 92.54	**<0.001** **^b^**
	6I	191.08 ± 71.82	220.48 ± 88.14	**<0.001** **^b^**
	6N	142.79 ± 47.88	170.51 ± 67.37	**<0.001** **^b^**

a *p* value determined by the Wilcoxon signed-rank test.

b *p* value determined by the paired *t*-test.

Significant *p* values (*p* < 0.05) are in bold.

1: central macular-foveal region; 3: parafoveal macular region; 6: perifoveal macular region; C: contralateral; MM: more myopic; T: temporal; N: nasal; S: superior; I: inferior.

### Comparison between retinal vascular density and choriocapillaris perfusion area

There was no statistical difference in the SVD, DVD, and CCPA of the MM and contralateral eyes in all areas according to the ETDRS (all *p* > 0.05) (Table 3).

**TABLE 3:** Comparison of the retinal vascular density and choriocapillaris perfusion area of more myopic and contralateral eyes.

		MM	Contralateral	P
Superficial vascular complex density (%)	1	7.26 ± 2.87	6.89 ± 2.63	0.623^b^
	3S	29.43 ± 7.10	27.38 ± 5.61	0.131^a^
	3T	24.33 ± 5.74	23.29 ± 5.30	0.327^a^
	3I	29.05 ± 7.71	27.93 ± 5.10	0.458^a^
	3N	27.79 ± 6.53	25.99 ± 6.32	0.202^a^
	6S	32.85 ± 6.46	33.24 ± 4.06	0.367^b^
	6T	24.03 ± 4.41	23.22 ± 3.75	0.348^b^
	6I	30.90 ± 6.58	32.21 ± 4.55	0.223^a^
	6N	40.00 ± 9.05	40.03 ± 7.17	0.986^a^
Deep vascular complex density (%)	1	10.81 ± 7.13	10.10 ± 5.92	0.879^a^
	3S	31.64 ± 12.03	31.46 ± 9.90	0.935^b^
	3T	32.42 ± 11.82	31.85 ± 10.83	0.701^a^
	3I	33.17 ± 11.61	30.95 ± 10.84	0.416^a^
	3N	35.09 ± 10.89	33.29 ± 9.17	0.408^b^
	6S	34.19 ± 11.08	35.20 ± 9.03	0.662^a^
	6T	38.63 ± 12.23	34.49 ± 12.67	0.091^a^
	6I	33.63 ± 11.55	32.50 ± 9.39	0.636^a^
	6N	35.77 ± 11.80	35.43 ± 9.53	0.636^a^
Choriocapillaris perfusion area (mm^2^)	1	0.47 ± 0.13	0.44 ± 0.14	0.225^b^
	3S	0.78 ± 0.19	0.74 ± 0.18	0.265^b^
	3T	0.74 ± 0.22	0.72 ± 0.20	0.640^b^
	3I	0.77 ± 0.20	0.72 ± 0.15	0.222^b^
	3N	0.73 ± 0.20	0.70 ± 0.18	0.467^b^
	6S	2.75 ± 0.53	2.70 ± 0.40	0.598^a^
	6T	2.67 ± 0.73	2.62 ± 0.60	0.733^b^
	6I	2.68 ± 0.62	2.53 ± 0.45	0.227^b^
	6N	2.52 ± 0.62	2.49 ± 0.50	0.807^b^

a *p* value determined by the Wilcoxon signed-rank test.

b *p* value determined by the paired *t*-test.

Significant *p* values (*p* < 0.05) are in bold.

1: central macular-foveal region; 3: parafoveal macular region; 6: perifoveal macular region; MM: more myopic; T: temporal; N: nasal; S: superior; I: inferior.

### Correlation between AL and macular structures

Non-parametric correlation analysis was used to explore the correlation between AL and macular structures. Spearman’s rank-order correlation coefficient was also calculated. AL was significantly correlated with the thickness of TRL, GCIPL, INL, ORL, NFL, and CHL (all *p* < 0.05) (Table 4).

**TABLE 4:** Analysis of correlations between the regional thickness of each retinal layer, choroidal layer, and axis length

		r_s_	95% CI	*p-value*
Total retinal layer (μm)	1	-0.252	-0.467∼0.014	0.041
	3S	-0.306	-0.518∼-0.072	**0.012**
	3T	-0.313	-0.525∼-0.103	**0.010**
	3I	-0.357	-0.560∼-0.116	**0.003**
	3N	-0.359	-0.561∼-0.125	**0.003**
	6S	-0.236	-0.467∼-0.013	0.057
	6T	-0.229	-0.446∼0.006	0.065
	6I	-0.395	-0.599∼-0.142	**0.001**
	6N	-0.383	-0.575∼-0.153	**0.001**
Ganglion cell-inner plexiform layer (μm)	1	-0.100	-0.332∼0.146	0.426
	3S	-0.147	-0.362∼0.096	0.239
	3T	-0.174	-0.379∼0.054	0.163
	3I	-0.251	-0.452∼-0.025	**0.042**
	3N	-0.314	-0.510∼-0.072	**0.010**
	6S	0.026	-0.219∼0.296	0.835
	6T	-0.194	-0.404∼0.027	0.118
	6I	-0.190	-0.433∼0.061	0.126
	6N	-0.216	-0.432∼0.039	0.082
Inner nuclear layer (μm)	1	-0.177	-0.421∼0.084	0.154
	3S	-0.417	-0.615∼-0.184	<**0.001**
	3T	-0.453	-0.643∼-0.225	<**0.001**
	3I	-0.428	-0.647∼-0.167	<**0.001**
	3N	-0.301	-0.489∼-0.069	**0.014**
	6S	-0.263	-0.488∼-0.010	**0.033**
	6T	-0.221	-0.458∼0.048	0.075
	6I	-0.198	-0.439∼0.052	0.110
	6N	-0.136	-0.404∼0.133	0.277
Outer retina layer (μm)	1	-0.402	-0.571∼-0.179	**0.001**
	3S	-0.338	-0.535∼-0.112	**0.006**
	3T	-0.301	-0.538∼-0.049	**0.014**
	3I	-0.252	-0.464∼-0.004	**0.041**
	3N	-0.415	-0.617∼-0.181	**0.001**
	6S	-0.372	-0.570∼-0.150	**0.002**
	6T	-0.242	-0.448∼-0.026	**0.050**
	6I	-0.347	-0.554∼-0.113	**0.004**
	6N	-0.492	-0.655∼-0.276	<**0.001**
Nerve fiber layer (μm)	1	0.581	0.390∼0.714	<**0.001**
	3S	0.280	0.040∼0.511	**0.023**
	3T	0.437	0.220∼0.606	<**0.001**
	3I	-0.048	-0.276∼-0.195	0.702
	3N	0.341	0.108∼0.530	**0.005**
	6S	0.163	-0.109∼0.399	0.191
	6T	0.271	0.032∼0.472	**0.028**
	6I	-0.160	-0.388∼-0.110	0.200
	6N	-0.070	-0.324∼0.183	0.578
Choroid layer (μm)	1	-0.522	-0.675∼-0.297	<**0.001**
	3S	-0.501	-0.674∼-0.287	<**0.001**
	3T	-0.537	-0.700∼-0.333	<**0.001**
	3I	-0.597	-0.744∼-0.403	<**0.001**
	3N	-0.513	-0.685∼-0.280	<**0.001**
	6S	-0.452	-0.629∼-0.222	<**0.001**
	6T	-0.546	-0.697∼-0.336	<**0.001**
	6I	-0.683	-0.793∼-0.519	<**0.001**
	6N	-0.526	-0.692∼-0.312	<**0.001**

Significant *p* values (*p* < 0.05) are in bold.

r_s_: Spearman’s rank-order correlation coefficient.

1: central macular-foveal region; 3: parafoveal macular region; 6: perifoveal macular region; T: temporal; N: nasal; S: superior; I: inferior.

CI: Confidence Interval.

## Discussion

The current study compared the changes in the intraretinal thickness, choroidal thickness, retinal vascular density, and CCPA of every subregion according to the grid of the ETDRS of highly myopic anisometropia. This study provides a new perspective for exploring alterations in macular structures and vascular characteristics with different degrees of high myopia. Furthermore, the effects of age, sex, environment, and genetics on macular structures and vascular characteristics of different individuals were excluded.

During our study, the TRL layers of the MM eyes were thinner in the perifoveal and parafoveal regions than in the contralateral eyes with highly myopic anisometropia. The TRL thickness was negatively correlated with AL. Using animal models of myopia, histological and *in vivo* OCT studies have demonstrated retinal thinning in myopic eyes, mainly caused by decreased areal cell density (Abbott et al., 2011). Furthermore, studies of the thickness of the retina in humans have demonstrated that the retina thinned in highly myopic eyes. Salehi et al. (Salehi et al., 2022) concluded that retinal thickness in the high myopia group was thinner than that in the emmetropia group in the parafoveal and perifoveal regions. Zhao et al. (Zhao et al., 2015) compared the retinal thickness with different severities of myopia and showed that the thickness of the retina became significantly thinner as the severity of myopia increased in the parafoveal and perifoveal regions. Our study also revealed that the TRL thickness in MM eyes was thinner than that in the contralateral eyes in most regions of the parafoveal and perifoveal rings. Hence, we hypothesized that the thickness of the retina thinned with the increase in the AL with high myopia.

Our study found that the GCIPL, INL, and ORL contributed the most to the total retinal thinning with the increased AL with high myopia. The GCIPL, INL, and ORL thicknesses were negatively correlated with AL. The same conclusions have been found during animal studies of myopia; ganglion cells and the inner and outer nuclear layers of the retina have been shown to decrease with the increase in AL with myopia (Zi et al., 2020). Moreover, as the AL increased with high myopia, the retinal thickness changes varied in different regions. Our study showed that more quadrants and layers were thinned in the MM eyes than in the contralateral eyes in the perifoveal regions, followed by those in the parafoveal regions, with no change in the foveal and temporal perifoveal regions. We hypothesized that retinal thinning is more pronounced in the peripheral zone as the AL increases; however, the peripheral temporal retina is unaffected. Some studies similar to ours also concluded that as the AL increased, retinal thickness decreased outside the foveal macular region with high myopia; furthermore, the retinal thickness also decreased in the perifoveal or parafoveal region, but not in the foveal region (Ikuno and Tano, 2009; Yamashita et al., 2013; Moon et al., 2021). This is probably because the population we studied had high myopia. There are significant regional differences in retinal growth that maintain the cellular density of the posterior pole, which is significantly important for maintaining BCVA (Lee et al., 2021). Moreover, with increased AL, the eye morphology mostly exhibits a longitudinal oval shape with a predominantly dilated posterior pole. Therefore, we hypothesized that layers of the intraretinal structures were subjected to greater strain in the posterior pole (nasal side of the macula), and the temporal side is less affected (Lee et al., 2021). Animal studies have also shown that temporal retinal thickness is less affected as the eye axis grows (Abbott et al., 2011).

We found a thinner GCIPL in MM eyes in the perifoveal region. A longitudinal study examined the changes in the GCILP thickness with the development of the AL with high myopia (Lee et al., 2020) and concluded that the GCIPL thickness of the high myopia group was thinner than of the emmetropes group in all regions, and that the GCIPL thinned with age. A similar conclusion was reported by Lu et al. (Lu et al., 2021), who further discovered that the thickness of the GCIPL was thinner in all regions in the myopic group. Our findings are consistent with those of previous studies; we found that the GCIPL in MM eyes was thinner than that in contralateral eyes. However, the GCIPL was mainly thinned in the perifoveal region during our study. This difference is possibly attributed to the different populations we studied. Previous studies compared the differences in the GCIPL thickness with myopes and emmetropes; however, our study compared the differences between MM and contralateral eyes with highly myopic anisometropia. The difference in the AL of the two groups in previous studies was larger than that observed during our study. During our study, the SE of the MM and contralateral eyes was higher than that of the myopic group observed during a previous study. In contrast, we used SS-OCT during our study, and the image magnification caused by the AL was corrected. Seo et al. (Seo et al., 2017) hypothesized that as the AL elongates, a larger retinal area leads to decreased macular ganglion cell complex density, which is more pronounced in the peripheral region. This may explain why the GCIPL was thinned mainly in the perifoveal region during our study. Experimental myopia models have shown that thinning of the INL and OPL contributes most to retinal thinning with myopic severity, followed by thinning of the photoreceptor cell layer and RPE (Abbott et al., 2011). *In vitro* studies have also shown thinning of the INL and ORL with AL elongation (Xin et al., 2021). Kirik et al. (Kirik et al., 2021) hypothesized that INL thinning in myopic eyes is caused by atrophy attributable to decreased activation of cells in the intraretinal layers. The INL in the MM eyes was thinner than that in the contralateral eyes in our study, consistent with the results of previous studies. Ye et al. (Ye et al., 2020) concluded that thinning of the deep retinal capillary complex led to thinning of the outer retina. More importantly, structural alterations in the ORL may lead to visual impairment and early visual field defects in patients with pathological myopia (Flores-Moreno et al., 2013; Wu et al., 2020). Therefore, it is important to explore the outer retinal thickness of patients with high myopia. Our findings further validated previous studies that reported thinning of the ORL occurs in many quadrants of the parafoveal and perifoveal regions in MM eyes. Notably, the NFL layer became thicker in the NFL-6S quadrant of the perifoveal layer in MM eyes in our study. The NFL thickness was positively correlated with AL. The thickness of the NFL in the nasal perifoveal quadrant of the macula did not show significant changes; however, it tended to become thicker in the MM eyes than in the contralateral eyes. The parafoveal NFL thickness showed no change in MM eyes compared to that in contralateral eyes, according to the study by Wang et al. (Wang et al., 2021) We suspect that this could be attributed to the growth of the AL with high myopia because the eye extending toward the posterior pole and vitreous can generate both tangential and perpendicular centrifugal forces (Parolini et al., 2021), resulting in an intensive centripetal force on the innermost layer of the retina, such as the ILM and RNFL. This may explain the increased thickness of the NFL in MM eyes in our study. Additionally, a previous study showed that with AL lengthening, the distribution of the RNFL thickness changed, which is related to retinal vascular displacement (Lee and Shields, 2010). With the growth of the AL with high myopia, the optic disc is tilted, the angle of the central retinal arterial trunk is decreased, and the central vascular trunk is stretched directionally to the nasal side of the optic disc (Lee et al., 2018; Lee et al., 2021). We hypothesized that the central retinal arterial trunk dragged the NFL from the nasal side to the temporal side of the optic disc, resulting in temporal thickening of the optic disc. All these changes may pull the NFL layer toward the superonasal area; therefore, we hypothesized that the NFL above the macula and on the nasal side will become thicker.

In addition to retinal thickness, the choroid supplies the outer retina with metabolically necessary substances and oxygen, which have an important role in maintaining normal vision. Choroidal thinning was remarkable in myopia, which preceded retinal thinning, and choroidal thickness was considered a better predictor of pathological alterations in myopic eyes than retinal thickness (Vincent et al., 2013; Jin et al., 2016; El-Shazly et al., 2017; Zhang et al., 2019). We compared nine regions of choroidal thickness of the MM and contralateral eyes with highly myopic anisometropia. Corresponding with previous results, our results showed thinner CHL in the MM eyes than in the contralateral eyes in all areas according to the ETDRS. The choroidal thickness was negatively correlated with AL. We hypothesized that the effect of AL lengthening on choroidal thickness is greater than the effect of AL lengthening on retinal thickness; however, longitudinal studies are required to confirm this.

OCTA was used during our study to quantitatively evaluate SVD and DVD in the macula. No significant differences between the MM and contralateral eyes were observed. This was in agreement with the results of the study by Yang et al. (Yang et al., 2017), who concluded that the macular vascular density of young, healthy adults was not influenced by the myopic severity. A study by Mo et al. (Mo et al., 2017) concluded that there were no significant changes in the retinal flow density of the macula of highly myopic and emmetropic eyes, which was consistent with our results. We hypothesized that the macular vascular density was not affected by the AL with high myopia. In contrast, a meta-analysis by Wang et al. (Wang et al., 2021) reported that whole superficial vessel density and deep vessel density were lower in HM than in control eyes, but foveal and parafoveal superficial vessel density showed no significant changes in HM compared to control eyes. We speculate that the different segmentation algorithms of OCTA brands led to the discrepancies. Fan et al. (Fan et al., 2017) concluded that the macular deep and superficial retinal vascular densities in the highly myopic group were lower than those in the control group. However, the highly myopic group had worse BCVA than the control group during their study, suggesting that the pathological changes caused by high myopia in their study affected retinal vascular densities. Mo et al. (Mo et al., 2017) also found that the flow density in the macula with high myopia with pathological changes was lower than that with high myopia without pathological changes and emmetropia. We hypothesized that the macular vascular density would decrease with pathological myopia accompanied by visual impairment. One of the exclusion criteria included in our study was BCVA <20/25, as confirmed by the Snellen eye chart. No significant differences in SVD and DVD in the macula between the MM and contralateral eyes were observed in our study. Additionally, during our study, we found no difference in the CCPA of the MM and contralateral groups. Jiang et al. (Jiang et al., 2021) and Wang et al. (Wang et al., 2021) also concluded that there were no significant differences in all sectors of the choroidal capillary layers of the high myopia and non-high myopia groups. Furthermore, Panda-Jonas et al. (Panda-Jonas et al., 2021) found that choriocapillaris thickness and density were not correlated with the AL. Angiographic studies have revealed that the choriocapillaris in highly myopic eyes with patchy atrophy exhibited a significant loss of choriocapillaris structure (Sayanagi et al., 2017). Our study excluded patchy atrophy changes, which might explain the lack of significant changes in the CCPA of the MM and contralateral eyes. Our study confirmed that highly myopic eyes with increased degrees of myopia are characterized mainly by changes in retinal thickness and that the status of the blood supply was unaffected. CHL thickness was significantly thinner in the MM eyes than in the contralateral eyes, but the CCPA did not exhibit any changes. This can explain why extremely thin choroidal thickness in some highly myopic eyes is compatible with good visual acuity (Pang et al., 2015). It is possible that although the choroidal thickness is significantly thinner, the CCPA can remain relatively unchanged, thus allowing the outer retina to receive a relatively adequate blood supply.

Our study has some limitations. First, this was a cross-sectional study. Longitudinal observations of the retinal and choroidal characteristics and the relationship between structure and function would help clarify the pathogenesis of pathological myopia. Second, the sample size was relatively small because of the low prevalence of myopic anisometropia (Afsari et al., 2013). A larger sample size study is required to verify the changes in macular structures and vascular characteristics. Third, because of the critical role of the ORL in the visual acuity of highly myopic eyes, we lacked sufficient specificity for stratification of the outer retina. The segmentation of the outer retina should be more specific in the future. In addition, the study group was rather homogeneous; therefore, further studies should include additional comparison groups with different age, sex, and refraction groups (with homogeneity of all the other factors).

In conclusion, OCTA is effective for the noninvasive and rapid evaluation of structural and vascular changes in HM. The intraretinal thickness, except for that of the RNFL layer, was thinner in the MM eyes than in the contralateral eyes with highly myopic anisometropia. The CHL thickness in the MM eyes was thinner in all regions of the rings according to the ETDRS. There were no statistical differences in the vascular complex density and CCPA of the MM and contralateral eyes. The causal relationship between these changes and the growth of the eye axis with high myopia needs to be further corroborated by longitudinal studies.

## Data Availability

The raw data supporting the conclusions of this article will be made available by the authors, without undue reservation.
